# Effect of Surface Adjustment and Hydrothermal Aging on the Mechanical Behavior and Microstructure of Zirconia With Different Yttria Contents

**DOI:** 10.1155/ijod/9739387

**Published:** 2026-08-25

**Authors:** Phawinee Phuntusuntorn, Natthawat Pleumsamran, Kullapop Suttiat

**Affiliations:** ^1^ Department of Prosthodontics, Faculty of Dentistry, Chiang Mai University, Chiang Mai 50200, Thailand, cmu.ac.th

**Keywords:** 3Y-TZP, 5Y-PSZ, 6Y-PSZ, biaxial flexural strength, hydrothermal aging, surface finishing and polishing

## Abstract

**Objectives:**

This in vitro study evaluated the effects of restorative surface adjustment and simulated hydrothermal aging on the microstructure and biaxial flexural strength of computer‐aided design (CAD)/CAM zirconia with different yttria contents, including conventional 3 mol% yttria‐stabilized tetragonal zirconia polycrystal (3Y‐TZP) and high‐translucency zirconia (5Y‐PSZ and 6Y‐PSZ).

**Methods:**

Disc‐shaped specimens (12 mm diameter × 1.2 mm thickness; *n* = 40 per material) were fabricated from zirconia blocks containing 3, 5, or 6 mol% yttria. Specimens were allocated to two surface conditions: control (no adjustment) or grinding with 100 µm diamond bur followed by a three‐step zirconia polishing system. Each condition was divided into aged and nonaged subgroups (*n* = 10). Hydrothermal aging was performed in an autoclave at 134°C and 0.2 MPa for 5 h as an accelerated low‐temperature degradation (LTD) process. Surface roughness and hardness were evaluated using a profilometer and nanoindentation tester, respectively. Surface morphology, grain size, and fracture characteristics were analyzed using scanning electron microscopy (SEM). Crystalline phase composition was evaluated using qualitative X‐ray diffraction (XRD). Biaxial flexural strength was measured via piston‐on‐three‐ball test (ISO 6872). Data were analyzed using three‐way analysis of variance (ANOVA) and Tukey’s test (*α* = 0.05).

**Results:**

Surface adjustment significantly reduced surface roughness. Biaxial flexural strength increased significantly in 3Y‐TZP and showed a less pronounced response in 5Y‐PSZ, whereas no significant differences were observed in 6Y‐PSZ. Hydrothermal aging did not significantly affect surface roughness, hardness, grain size, or biaxial flexural strength under the applied conditions. No monoclinic reflections were detected by qualitative XRD. However, SEM revealed localized grain‐boundary microcracking and surface degradation in aged 3Y‐TZP.

**Conclusion:**

Finishing and polishing (FP) improved the biaxial flexural strength of 3Y‐TZP and moderately enhanced that of 5Y‐PSZ, whereas 6Y‐PSZ showed limited sensitivity to surface adjustment. Simulated hydrothermal aging did not significantly compromise the mechanical performance or phase stability of the evaluated zirconia.

## 1. Introduction

Metal‐free restorative materials have become widely adopted in contemporary dentistry because of their favorable esthetics, excellent biocompatibility, and high mechanical strength. Among these materials, zirconia‐based ceramics—particularly yttria‐stabilized tetragonal zirconia polycrystal (Y‐TZP)—are extensively used for single crowns and fixed dental prostheses due to their superior fracture resistance and structural reliability [1, 2]. Conventional monolithic zirconia containing 3 mol% Y‐TZP (3Y‐TZP) exhibits flexural strength values that often exceed 1000 MPa, making it suitable for restorations subjected to substantial occlusal loading [3]. The high strength of 3Y‐TZP is primarily attributed to the transformation‐toughening mechanism, in which stress concentration at a crack tip triggers a phase transformation from the metastable tetragonal phase to the monoclinic phase. This transformation is accompanied by a localized volumetric expansion that generates compressive stresses around the crack, thereby inhibiting crack propagation and enhancing fracture resistance [4].

Despite these mechanical advantages, conventional 3Y‐TZP demonstrates limited translucency and relatively high opacity, which restrict its ability to reproduce the optical characteristics of natural teeth, particularly in the esthetic anterior region [5, 6].

To overcome this limitation, newer zirconia materials with increased yttria (Y_2_O_3_) content have been developed to enhance the translucency [7, 8]. Increasing the yttria concentration stabilizes a greater proportion of the cubic phase within the zirconia microstructure. Because the cubic phase is optically isotropic and lacks birefringence, it reduces light scattering at grain boundaries and thereby improves the optical transmission of the material [3, 9]. Furthermore, multilayer zirconia systems have been introduced to mimic the natural gradient of tooth structure by incorporating increased opacity and chroma in the cervical region and greater translucency in the incisal region, thereby reproducing the enamel–dentin architecture of natural teeth [10].

However, improvements in optical properties are frequently accompanied by compromises in mechanical performance. Increasing the yttria content reduces the fraction of transformable tetragonal grains, thereby weakening the transformation‐toughening mechanism responsible for the high fracture resistance of conventional zirconia [11, 12]. Consequently, high‐translucency zirconia materials‐particularly those containing 5 mol% yttria (5Y‐PSZ) generally exhibit lower flexural strength and fracture toughness compared with conventional 3Y‐TZP [11].

In clinical practice, zirconia restorations frequently require occlusal adjustment during try‐in or delivery to achieve accurate occlusal contacts and proper prosthesis seating. Such adjustments are typically performed using diamond burs, followed by polishing procedures. These interventions inevitably modify the surface characteristics of zirconia and may introduce surface defects, residual stresses, or phase transformations that could influence the mechanical behavior of the material. Previous investigations have demonstrated that grinding procedures may induce tetragonal‐to‐monoclinic phase transformation in 3Y‐TZP and lead to a reduction in flexural strength, particularly when coarse diamond instruments are used without adequate polishing [13]. For this reason, polishing with zirconia‐specific diamond polishing systems has been recommended to restore surface smoothness and maintain the mechanical integrity of zirconia restorations.

Nevertheless, the influence of surface finishing procedures remains controversial. Some studies have reported that fine polishing may introduce surface microcracks or cause surface embrittlement, potentially reducing the fracture toughness [14]. In addition, localized heat generation during grinding may promote thermally induced phase transformation when the temperature exceeds the critical threshold required for the tetragonal‐to‐monoclinic transformation, which may further contribute to the reduction in the flexural strength of zirconia restorations following surface adjustment [15].

Beyond these immediate mechanical effects, zirconia restorations are continuously exposed to a humid oral environment with fluctuating temperatures. Under such conditions, zirconia may undergo low‐temperature degradation (LTD), a phenomenon characterized by progressive tetragonal‐to‐monoclinic phase transformation in the presence of water vapor [16, 17]. Surface defects created during grinding and polishing may facilitate water penetration and accelerate the initiation and propagation of LTD, resulting in microstructural changes that may compromise the long‐term mechanical reliability of zirconia restorations [18, 19]. Therefore, evaluating the combined effects of restorative surface adjustment and hydrothermal aging is essential for predicting the long‐term performance of zirconia materials under clinically relevant conditions.

Although the effects of grinding and polishing have been extensively studied in conventional 3Y‐TZP, limited information is available regarding how these procedures influence the mechanical behavior and microstructural stability of newer high‐translucency zirconia materials with higher yttria contents. Given the increasing clinical use of these materials, a better understanding of their response to surface adjustment and aging conditions is necessary.

Therefore, the aim of this in vitro study was to evaluate the effect of restorative surface adjustment on the microstructure and biaxial flexural strength of zirconia with different yttria contents, including conventional 3Y‐TZP and high‐translucency zirconia represented by 5Y‐PSZ and 6Y‐PSZ. In addition, simulated hydrothermal aging was performed to estimate their long‐term performance under intraoral environmental conditions and to assess the stability of these materials following aging‐related degradation. The null hypotheses were that zirconia composition, restorative surface adjustment, hydrothermal aging, and their interactions would not affect surface roughness, surface hardness, grain size, or biaxial flexural strength; and that no changes in crystalline phase composition would be detected within the limits of the qualitative X‐ray diffraction (XRD) analysis used.

## 2. Materials and Methods

### 2.1. Materials

Dental zirconia materials with 3%, 5%, and 6% yttria stabilizer content were selected to represent different categories of contemporary zirconia used in clinical practice. Conventional monolayer zirconia was represented by 3Y‐TZP (DD BioZX2 Zirconia), while the high‐translucency multilayer zirconia were represented by 5Y‐PSZ (DD cubeX2 Zirconia) and 6Y‐PSZ (Nacera Pearl Natural Zirconia). All materials were manufactured by Dental Direkt (Spenge, Germany). The material compositions and specifications are summarized in Table 1.

**Table 1:** Manufacturer’s specifications and chemical compositions of selected materials.

Group	Material	Lot. no.	Chemical compositions (wt.%)
3Y‐TZP	DD Bio ZX2 Zirconia	6162221001	ZrO_2_ + HfO_2_ + Y_2_O_3_ ≥ 99.0 Y_2_O_3_ < 6 Al_2_O_3_ < 0.15 other oxides < 1.0
5Y‐PSZ	DD Cube X2 Zirconia	1162118015	ZrO_2_ + HfO_2_ + Y_2_O_3_ ≥ 99.0 Y_2_O_3_ ≤ 10 Al_2_O_3_ ≤ 0.15 other oxides < 1.0
6Y‐PSZ	Nacera Pearl Natural Zirconia	6162308023	ZrO_2_ + HfO_2_ + Y_2_O_3_ ≥ 99.0 Y_2_O_3_ < 9 Al_2_O_3_ ≤ 0.1 other oxides < 1.0

### 2.2. Specimen Preparation

#### 2.2.1. Specimen Fabrication

The standard tessellation language (STL) file of a zirconia rod with a circular cross‐section 15 mm in diameter and 16 mm in length, oversized by approximately 20% to compensate for sintering shrinkage as recommended by the manufacturers, was generated using computer‐aided design (CAD) software (3Shape Dental System; 3Shape A/S). Partially sintered zirconia discs of 3Y‐TZP, 5Y‐PSZ, and 6Y‐PSZ were milled into rod‐shaped specimens using a 5‐axis dental milling machine (Xmill 500 Plus, Dental Direkt, Spenge, Germany).

The milled specimens fabricated from 3Y‐TZP and 5Y‐PSZ were sintered at 1500°C for 120 min, whereas the 6Y‐PSZ specimens were sintered at 1450°C for 90 min, in accordance with the manufacturers’ recommendations. After complete sintering, the zirconia rods were carefully finished and measured to verify the final dimensions of 12 mm in diameter and 13 mm in length using a digital caliper with a resolution of 0.001 mm (TorQ 150 x 0.001 mm Digital Caliper, China).

For each zirconia type (3Y‐TZP, 5Y‐PSZ, and 6Y‐PSZ), 40 disc‐shaped specimens were prepared for biaxial flexural strength testing in accordance with ISO 6872:2015. The fully sintered zirconia rods were sectioned into discs measuring 12 mm in diameter and 1.2 ± 0.2 mm in thickness using a slow‐speed diamond cutting disc mounted on a precision cutting machine (Isomet 1000 Precision Saw, Buehler, Lake Bluff, IL, USA) under continuous water irrigation. The final dimensions of each specimen were confirmed using the same digital caliper to ensure standardization and a constant thickness of exactly 1.2 mm for all final specimens.

#### 2.2.2. Surface Treatment

All disc specimens free of visible defects were subjected to standardized surface smoothing using sequential silicon carbide abrasive papers (600, 800, and 1200 grit) under continuous water irrigation. Polishing was performed using an automatic polishing machine (Struers LaboPol‐20 grinding and polishing machine, Denmark) operating at 300 rpm with an applied pressure of 1 N/cm^2^, with each polishing step lasting 60 s. After surface smoothing, all specimens were then ultrasonically cleaned in distilled water at 37°C for 5 min and air‐dried, and their surface roughness was verified using a contact profilometer (Surftest SJ‐301, Mitutoyo, Tokyo, Japan).

The intact specimens from each zirconia type were randomly allocated into two subgroups (*n* = 20/group) according to surface adjustment procedures: specimens without surface adjustment (G1) and specimens subjected to surface adjustment (G2). Each subgroup was further divided into aged and nonaged conditions (*n* = 10/group). The sample size of 10 specimens per experimental group (*n* = 10, total *N* = 120) was selected in accordance with the ISO 6872:2015 standard (dentistry‐ceramic materials), which specifies a minimum of 10 specimens per group for biaxial flexural strength testing. The sample size was determined prior to the experiment using *G* ^∗^Power software (version 3.1.9.7). Based on an expected effect size of *f* = 0.40, a significance level of *α* = 0.05, and a desired statistical power of 80% across the 12 experimental conditions, a total sample size of *N* = 120 was calculated to be sufficient for detecting significant differences. For the surface adjustment group (G2), specimens were ground using a 100 µm diamond bur (881Z4, Meisinger, Neuss, Germany) in a sweeping forward–backward motion at 200,000 rpm for 20 s. Subsequently, specimens were sequentially polished using a three‐step zirconia polishing system (ZiLMaster, Shofu, San Marcos, CA, USA) following the same motion, with each polishing step performed for 1 min. This step simulated the prosthesis adjustment during the clinical procedure. A straight electric handpiece (A‐Dec EA‐51LT, A‐Dec, Newberg, OR, USA) was positioned parallel to the specimen surface by attaching to the fixed holder arm during the procedure to ensure consistent pressure, orientation, and polishing direction of surface adjustment. Then, the adjusted specimens were ultrasonically cleaned in distilled water at 37°C for 5 min to remove the residual particles of polishing burs and the ground off zirconia and air‐dried.

#### 2.2.3. Simulated Hydrothermal Aging

To induce LTD, half of the specimens from both G1 and G2 groups for each zirconia type (*n* = 10/group) were subjected to hydrothermal aging in an autoclave (Model 1730 MK) following ISO 13356:2015 at 134°C under a pressure of 0.2 MPa for 5 h. According to Chevalier et al. [20], 1 h of steam sterilization at 134°C in an autoclave is equivalent to 3–4 years of aging in vivo.

### 2.3. Specimen Evaluations

#### 2.3.1. Biaxial Flexural Strength

Biaxial flexural strength was evaluated using a piston‐on‐three‐ball test in accordance with ISO 6872:2015 (*n* = 10/group). Three stainless‐steel support balls (3.2 mm in diameter) were positioned 120° apart on a circular metallic support with a diameter of 10 mm. Each specimen was centrally placed on the support balls with the finishing and polishing (FP) surface oriented facing downward, facing the tensile side, and was centrally loaded using a flat‐ended piston with a diameter of 1.4 mm. All tests were performed at a crosshead speed of 0.5 mm/min using a universal testing machine (Instron 5566, Norwood, MA, USA) until fracture occurred. The maximum load at failure (*P*, *N*) was recorded and converted to biaxial flexural strength (*S*, MPa) using the following equation (ISO 6872:2015):

S=−0.2387PX−Yd2,

where

X=1+νlnr2r32+1−ν2r2r32Y=1+ν1+lnr1r32+1−νr1r32.

In these equations, *S* is the maximum tensile stress at the center of the specimen (MPa), *P* is the fracture load (N), *ν* is Poisson’s ratio (0.25), *d* is the specimen thickness (1.2 mm), *r*_1_ is the radius of the support circle (5.0 mm), *r*_2_ is the radius of the loading piston (0.7 mm), and *r*_3_ is the radius of the specimen (6.0 mm).

After completion of the piston‐on‐three‐ball test, fractured specimens were collected, and representative samples were randomly selected for subsequent fractographic analysis.

### 2.4. Fracture Surface Analysis

Following biaxial flexural strength testing, 10 fractured specimens were randomly selected from each group for fractographic evaluation. Fracture surfaces were examined using scanning electron microscopy (SEM) (JSM‐IT800, JEOL, Tokyo, Japan) after gold sputter coating. SEM observations were performed at magnifications of × 1000 and × 4000 under low‐vacuum conditions (10–50 Pa) with an accelerating voltage of 15 kV and an emission current of 93 μA.

### 2.5. Surface Roughness and Morphology

The surface roughness of each specimen was measured using a contact stylus profilometer (Surftest SJ‐301, Mitutoyo, Tokyo, Japan). Three measurements were taken at different, randomly selected locations on each specimen surface, and the arithmetic mean roughness (Ra) value was calculated as the average of the three readings. The mean Ra value (µm) was used for statistical comparison among groups.

In addition, one representative specimen from each group was prepared for surface morphology evaluation using a SEM (JSM‐IT800, JEOL, Tokyo, Japan) at a magnification of × 2000.

### 2.6. Surface Hardness

Surface hardness was evaluated using a nanoindentation technique with a dynamic ultramicro hardness tester (DUH‐211, Shimadzu, Kyoto, Japan) (*n* = 5/group). Indentation measurements were performed on the polished surface of each specimen using a Berkovich indenter. For each specimen, three indentations were made in the central area of the disc, and the mean hardness value was calculated. A load was applied at a constant loading rate of 14 mN/s until the preset maximum load of 2940 mN was reached. The maximum load was held for 15 s before being unloaded at the same rate. Indentation hardness values were calculated using integrated software provided by the manufacturer (DUH‐211, Shimadzu, Kyoto, Japan).

### 2.7. Crystalline Structure

Ten specimens from each group were randomly selected from those subjected to the piston‐on‐three‐ball test for crystalline phase analysis. XRD measurements were performed on the intact external surface of the fragments using a diffractometer (D8 ADVANCE, Bruker, Germany) with Cu‐Kα radiation (λ = 1.5406 Å), operated at 40 kV and 40 mA. Diffraction patterns were collected over a 2θ range of 20°–80°, with a step size of 0.013°, and a scanning rate of 1°/min.

Phase identification was performed qualitatively by analyzing the diffraction patterns. The presence of monoclinic (m), tetragonal (t), and cubic (c) zirconia phases was identified by comparing the peak positions and relative intensities with standard JCPDS database cards.

### 2.8. Grain Size

Five specimens from each zirconia group were randomly selected from both aged and nonaged conditions for grain size evaluation. To enhance grain boundary visibility, the specimens were thermally etched at 1400°C for 30 min and then allowed to cool naturally to room temperature. The polished surfaces were subsequently gold‐sputter‐coated prior to examination.

Grain morphology and size were analyzed using SEM (JSM‐IT800, JEOL, Tokyo, Japan). SEM micrographs were acquired at a magnification of × 10,000. For each specimen, three images were obtained from different, randomly selected areas. Grain size measurements were performed using ImageJ software by analyzing at least 300 grains per specimen. The mean grain size, grain size distribution, and cumulative frequency were calculated using Origin software.

### 2.9. Statistical Analysis

Statistical analyses were performed using IBM SPSS Statistics, version 30.0 (IBM Corp., Armonk, NY, USA). For biaxial flexural strength, surface roughness, surface hardness, and grain size, a three‐way analysis of variance (ANOVA) was conducted with the zirconia material, surface condition, and aging condition as the independent fixed factors. The main effects and all interaction effects were examined. When indicated, Tukey’s honestly significant difference (HSD) test was used for multiple comparisons. The normality of data distribution was assessed using the Shapiro–Wilk test. The level of significance was set at *p* < 0.05.

## 3. Results

### 3.1. Surface Morphology and Surface Roughness

Three‐way ANOVA revealed that zirconia type, FP, and hydrothermal aging all exerted statistically significant main effects on surface roughness (*p* < 0.05, $\eta_{p}^{2}=0.78$, 0.94, and 0.33, respectively). However, no significant interaction effects were observed among these factors (*p* > 0.05).

Under control conditions, all zirconia materials exhibited comparable surface roughness values, with no statistically significant differences among groups (*p*  > 0.05) (Table 2). Although 6Y‐PSZ showed a slightly higher mean Ra than 3Y‐TZP and 5Y‐PSZ (95% CI: 0.18 and 0.20 µm), the magnitude of this difference was minimal and within experimental variability. SEM revealed irregular surface features characterized by machining‐related scratches and a relatively coarse surface texture across all zirconia types. Microstructural observations highlighted clear differences associated with yttria content: 3Y‐TZP exhibited a fine and homogeneous grain structure, whereas 5Y‐PSZ and 6Y‐PSZ showed a bimodal grain‐size distribution composed of smaller and larger grains.

**Table 2:** Mean values with standard deviation and statistical analysis of grain size, surface roughness, and surface hardness.

Zirconia	Group	Mean ± SD			
		Large grain size (µm)	Small grain size (µm)	Surface roughness (µm)	Surface hardness (GPa)
3Y‐TZP (DD BioZX2)	Control (C)	0.17 ± 0.001^a^	0.17 ± 0.001^ab^	0.21 ± 0.005^de^	13.01 ± 0.09
	Control + aging (CA)	0.17 ± 0.004^a^	0.17 ± 0.004^b^	0.21 ± 0.007^ef^	12.92 ± 0.04
	Polishing (FP)	0.16 ± 0.003^a^	0.16 ± 0.003^a^	0.13 ± 0.004^a^	13.32 ± 0.04
	Polishing + aging (FPA)	0.17 ± 0.001^a^	0.17 ± 0.001^ab^	0.14 ± 0.002^a^	13.28 ± 0.03
5Y‐PSZ (DD cubeX2)	Control (C)	0.81 ± 0.002^bc^	0.35 ± 0.003^d^	0.21 ± 0.002^ef^	13.76 ± 0.09
	Control + aging (CA)	0.83 ± 0.016^cd^	0.37 ± 0.003^e^	0.22 ± 0.002^ef^	13.17 ± 0.09
	Polishing (FP)	0.77 ± 0.053^b^	0.35 ± 0.003^c^	0.15 ± 0.012^ab^	13.82 ± 0.10
	Polishing + aging (FPA)	0.82 ± 0.003^bc^	0.36 ± 0.002^e^	0.18 ± 0.008^bc^	13.40 ± 0.02
6Y‐PSZ (Nacera)	Control (C)	0.90 ± 0.004^ef^	0.42 ± 0.007^g^	0.22 ± 0.012^ef^	13.97 ± 0.04
	Control + aging (CA)	0.94 ± 0.016^f^	0.44 ± 0.005^h^	0.24 ± 0.014^f^	13.37 ± 0.09
	Polishing (FP)	0.88 ± 0.009^de^	0.40 ± 0.004^f^	0.18 ± 0.001^bc^	14.14 ± 0.07
	Polishing + aging (FPA)	0.92 ± 0.006^ef^	0.44 ± 0.003^h^	0.18 ± 0.007^cd^	14.11 ± 0.02

*Note:* Different superscript letters in the same column indicate statistically significant differences (*p*  < 0.05).

FP resulted in a pronounced and statistically significant reduction in surface roughness for all zirconia materials compared with their respective control groups (*p*  < 0.05) (Table 2). Among the polished specimens, 3Y‐TZP demonstrated the lowest Ra values, followed by 5Y‐PSZ and 6Y‐PSZ. SEM analysis corroborated these findings, revealing smoother surfaces with markedly reduced scratch patterns and a more uniform surface appearance following polishing, irrespective of the zirconia composition.

After hydrothermal aging, a slight increase in surface roughness was observed in all polished groups; however, these changes were not statistically significant compared with the nonaged, polished specimens (*p*  > 0.05) (Table 2). The surface morphology of aged 5Y‐PSZ and 6Y‐PSZ remained largely unchanged, indicating good resistance to aging‐related surface alterations. In contrast, aged 3Y‐TZP specimens exhibited early microstructural features associated with LTD, including grain‐boundary microcracking and localized grain pull‐out, despite the absence of a significant change in mean roughness values.

### 3.2. Fracture Surface

**Figure 1:**
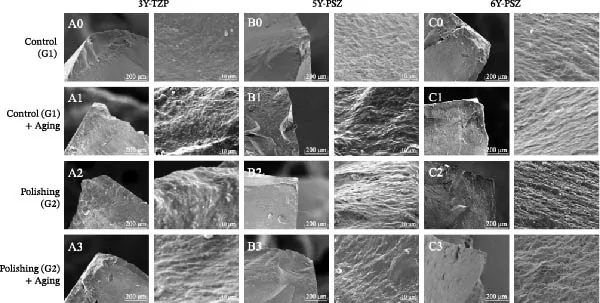
Representative SEM images of fracture surface morphology of zirconia specimens under different experimental conditions: control group of 3Y‐TZP (A0), control group after ageing of 3Y‐TZP (A1), finishing and polishing of 3Y‐TZP (A2), finishing and polishing after ageing of 3Y‐TZP (A3), control group of 5Y‐PSZ (B0), control group after ageing of 5Y‐PSZ (B1), finishing and polishing of 5Y‐PSZ (B2), finishing and polishing after ageing of 5Y‐PSZ (B3), control group of 6Y‐PSZ (C0), control group after ageing of 6Y‐PSZ (C1), finishing and polishing of 6Y‐PSZ (C2), and finishing and polishing after ageing of 6Y‐PSZ (C3). For each condition, low‐magnification images (200 µm scale bar) illustrate the overall fracture surface morphology, while higher‐magnification images (10 µm scale bar) reveal detailed microstructural features associated with crack propagation. The fracture surfaces exhibit typical brittle fracture characteristics with features such as fracture steps, hackle markings, and crack propagation patterns. No apparent morphological differences indicative of severe microstructural degradation was observed among the experimental conditions.

Representative SEM images of the fracture surfaces for all zirconia specimens are presented in Figure 1. The micrographs illustrate the fracture characteristics of 3Y‐TZP, 5Y‐PSZ, and 6Y‐PSZ under four experimental conditions: control, hydrothermal aging, and FP, followed by hydrothermal aging.

In the control groups, the fracture surfaces of all zirconia materials exhibited typical brittle fracture features. The 3Y‐TZP specimens (A0) displayed relatively rough fracture surfaces characterized by an irregular topography and pronounced fracture steps. At higher magnification, the surface revealed fine granular features and localized hackle patterns, indicating crack propagation through a polycrystalline microstructure. The fracture surfaces of 5Y‐PSZ (B0) and 6Y‐PSZ (C0) appeared comparatively smoother, with more uniform fracture planes and fewer pronounced ridges than those observed in 3Y‐TZP.

After hydrothermal aging, the fracture surface morphology remained generally similar to that observed in the control condition. In aged 3Y‐TZP specimens (A1), slightly increased surface roughness and more pronounced crack propagation markings were observed. The fracture surfaces exhibited distinct cleavage‐like facets and micro‐steps, suggesting crack deflection during fracture propagation. In contrast, the aged 5Y‐PSZ (B1) and 6Y‐PSZ (C1) specimens maintained relatively uniform fracture surfaces with limited evidence of microstructural alteration.

Specimens subjected to FP (A2, B2, and C2) demonstrated fracture surfaces that remained consistent with brittle fracture behavior. For 3Y‐TZP (A2), the fracture surfaces exhibited irregular ridges and micro‐roughness, similar to those observed in the control group. In the case of 5Y‐PSZ (B2) and 6Y‐PSZ (C2), the fracture surfaces appeared comparatively smoother, with more planar regions and fewer pronounced crack deflection features.

For specimens that underwent FP followed by hydrothermal aging (A3, B3, and C3), the overall fracture morphology remained comparable to the polished‐only condition. The 3Y‐TZP specimens (A3) continued to exhibit rough fracture surfaces with visible fracture steps and crack propagation features. Meanwhile, the fracture surfaces of 5Y‐PSZ (B3) and 6Y‐PSZ (C3) remained relatively smooth and homogeneous.

Overall, the SEM observations revealed that all zirconia materials exhibited typical brittle fracture characteristics regardless of the surface treatment or aging condition. Although minor variations in surface roughness and fracture features were observed, particularly in 3Y‐TZP, no distinct morphological changes indicative of severe surface degradation or microstructural damage were detected after finishing, polishing, or hydrothermal aging. These observations suggest that the applied surface treatments and aging conditions did not substantially alter the fracture behavior of the zirconia ceramics.

### 3.3. Surface Hardness

Three‐way ANOVA indicated that only zirconia type exerted a statistically significant main effect on surface hardness (*p* < 0.05, $\eta_{p}^{2}=0.25$), whereas FP ($\eta_{p}^{2}=0.08$), and hydrothermal aging ($\eta_{p}^{2}=0.07$), did not significantly affect the hardness values (*p* > 0.05). Furthermore, no significant interaction effects were observed among any of these factors (*p* > 0.05).

Nanoindentation testing revealed that surface hardness values were broadly comparable across all zirconia materials and experimental conditions. Although a gradual increase in mean hardness was observed with increasing yttria content (95% CI: 1328.97, 1375.24 MPa), this trend did not reach statistical significance (*p*  > 0.05). FP procedures did not produce measurable changes in surface hardness within any zirconia group. Similarly, hydrothermal aging had no significant effect on hardness values, regardless of the zirconia composition.

Overall, surface hardness remained stable across all zirconia types and treatment conditions, indicating that variations in the yttria content, as well as surface finishing and aging protocols, did not adversely affect the intrinsic resistance of the materials to localized plastic deformation.

### 3.4. Biaxial Flexural Strength

**Figure 2:**
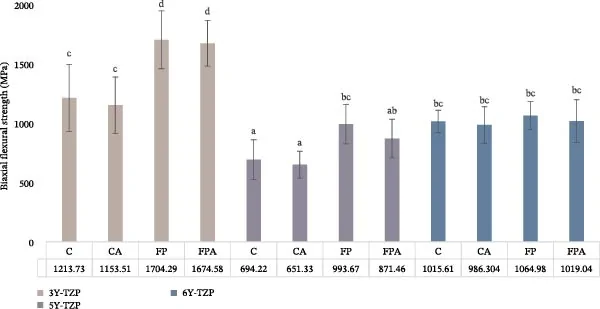
Average biaxial flexural strength and standard deviation of each zirconia material: 3Y‐TZP, 5Y‐PSZ, and 6Y‐PSZ. Different superscript letters above bars indicate significant differences between groups (*p*  < 0.05).

The biaxial flexural strength values of the zirconia materials under different surface conditions are presented in Figure 2. The results demonstrate significant differences among zirconia compositions and surface treatments.

Three‐way ANOVA revealed that zirconia type and FP exerted statistically significant main effects on biaxial flexural strength (*p* < 0.05, $\eta_{p}^{2}=0.69$ and 0.37, respectively), whereas hydrothermal aging did not (*p* > 0.05, $\eta_{p}^{2}=0.02$). Additionally, a statistically significant interaction effect was observed between zirconia type and FP (*p* < 0.05, $\eta_{p}^{2}=0.23$), while all other interaction effects were not statistically significant (*p* > 0.05).

For 3Y‐TZP, the control group exhibited a mean biaxial flexural strength of 1213.7 MPa, while hydrothermal aging slightly reduced the strength to 1153.5 MPa, although this difference was not statistically significant (*p*  > 0.05). Specimens subjected to FP showed the highest strength value (1704.3 MPa), which was significantly greater than both the control and aged groups (*p*  < 0.05). A similar trend was observed for specimens that underwent FP, followed by hydrothermal aging (FPA), with a mean strength of 1674.6 MPa, which remained significantly higher than the untreated groups. These findings indicate that FP substantially improved the strength of 3Y‐TZP and that subsequent aging did not markedly reduce the enhanced mechanical performance.

In contrast, 5Y‐PSZ demonstrated considerably lower strength values compared with those of 3Y‐TZP across all experimental conditions. The control and aged groups showed mean strengths of 694.2 MPa and 651.3 MPa, respectively, with no statistically significant difference between them. Surface FP increased the strength to 993.7 MPa, while specimens subjected to FP, followed by aging, exhibited a mean strength of 871.5 MPa. Although polishing resulted in higher strength values compared with those of the untreated groups, the increase was moderate relative to that observed in 3Y‐TZP.

For 6Y‐PSZ, the control specimens exhibited a mean biaxial flexural strength of 1015.6 MPa. Hydrothermal aging produced a slight reduction in the strength (986.3 MPa), which was not statistically significant. FP increased the mean strength to 1065 MPa, while FP, followed by aging, yielded a similar value (1019 MPa). Overall, the strength values of 6Y‐PSZ remained relatively stable across all surface conditions, with only minor variations being observed.

When comparing the three zirconia compositions, 3Y‐TZP exhibited the highest biaxial flexural strength, particularly after FP (overall 95% CI: 1022.01 and 1151.77 MPa), whereas 5Y‐PSZ showed the lowest strength values under all conditions. 6Y‐PSZ demonstrated intermediate strength values, consistently higher than those of 5Y‐PSZ but lower than those of the polished 3Y‐TZP specimens.

Overall, FP significantly increased the biaxial flexural strength of zirconia, particularly in 3Y‐TZP, while hydrothermal aging alone had minimal influence on the strength. These results indicate that surface finishing procedures may enhance the mechanical performance of zirconia restorations, whereas the simulated aging conditions applied in this study did not substantially compromise the structural integrity of the tested materials.

### 3.5. Phase Composition Analysis by XRD

**Figure 3:**
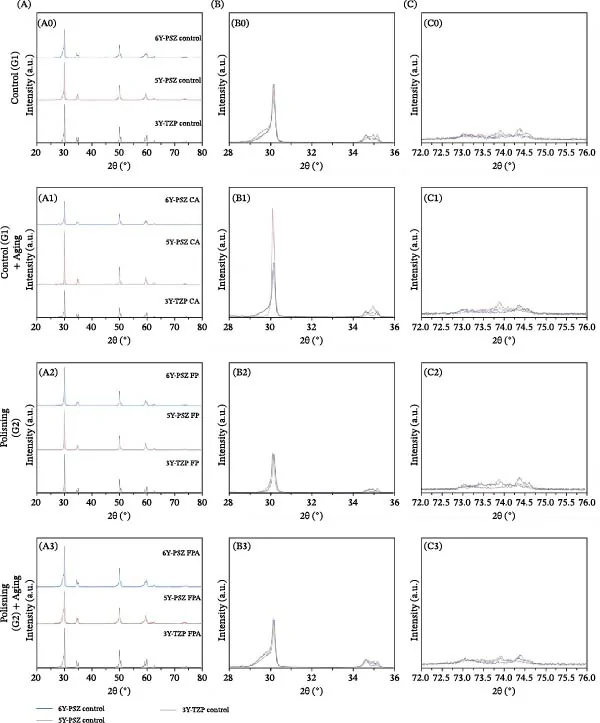
X‐ray diffraction (XRD) patterns of zirconia with different yttria contents under various surface conditions. Representative XRD diffractograms of 3Y‐TZP, 5Y‐PSZ, and 6Y‐PSZ are shown for four experimental conditions: as‐sintered control (A0–C0), hydrothermal aging (A1–C1), grinding with 100 μm diamond bur and polishing with a three‐step zirconia polishing system (A2–C2), and grinding/polishing followed by hydrothermal aging (A3–C3). For each condition, three angular regions are presented: (A) full diffraction range (20°–80° 2θ), (B) magnified principal peak region (28°–36° 2θ), and (C) high‐angle region (72°–76° 2θ). The diffraction patterns demonstrate phase characteristics typical of yttria‐stabilized zirconia. Across all materials and experimental conditions, no distinct monoclinic reflections were detected near 28.2° or 31.5° (2θ).

Representative XRD patterns of all zirconia materials under different surface conditions are shown in Figure 3. The diffractograms are presented in three angular regions: the full diffraction range (20°–80° 2θ), the principal peak region (28°–36° 2θ), and the high‐angle region (72°–76° 2θ). These regions are frequently examined in zirconia research because they allow identification of the main crystallographic phases and enable detection of possible tetragonal‐to‐monoclinic transformation associated with LTD [21, 22].

In the as‐sintered condition, all materials exhibited diffraction patterns typical of yttria‐stabilized zirconia ceramics. The XRD pattern of 3Y‐TZP showed well‐defined peaks at approximately 30°, 35°, 50°, and 60° (2θ), corresponding to the [101, 110, 112, and 211] reflections of the tetragonal phase. In contrast, the diffraction patterns of 5Y‐PSZ and 6Y‐PSZ presented broader peaks and reduced asymmetry in the principal peak region. This feature is consistent with the presence of a mixed tetragonal–cubic microstructure resulting from the higher yttria content in these materials. Within the limits of the qualitative XRD analysis used, no distinct reflections corresponding to the monoclinic phase were detected in any of the materials in the as‐sintered state.

After hydrothermal aging, the overall diffraction profiles of all zirconia compositions remained similar to those of the as‐sintered condition. In the magnified peak region near 30° (2θ), minor variations in peak intensity and peak width were observed in the 3Y‐TZP specimens, although the peak positions remained unchanged. The diffraction patterns of 5Y‐PSZ and 6Y‐PSZ exhibited negligible differences following aging. Importantly, no monoclinic diffraction peaks were detected near 28.2° or 31.5° (2θ), within the limits of qualitative XRD analysis, indicating that there was no detectable evidence of significant tetragonal‐to‐monoclinic phase transformation under the simulated aging conditions.

Following restorative surface adjustment by grinding and subsequent polishing using a three‐step zirconia polishing system, the diffraction patterns of all materials remained largely comparable to those of the control condition. The principal diffraction peaks were located at the same angular positions across all zirconia compositions. However, slight peak broadening was observed in the magnified region between 28° and 36° (2θ), particularly in specimens that underwent grinding. This broadening may indicate the presence of lattice strain or residual surface stresses introduced by the mechanical adjustment procedure. Despite this observation, no additional peaks associated with the monoclinic phase were detected within the limits of the qualitative XRD analysis used.

For specimens that underwent grinding and polishing, followed by hydrothermal aging, the XRD patterns remained like those observed in the polished‐only condition. Minor peak broadening persisted in the principal peak region for 3Y‐TZP, which may reflect the residual lattice strain induced during grinding. In contrast, the diffraction profiles of 5Y‐PSZ and 6Y‐PSZ remained essentially unchanged. Hydrothermal aging after restorative surface adjustment did not produce peak splitting or measurable peak shifts in any material, indicating that the crystallographic structures remained stable. Overall, the results suggest that neither the FP procedures nor the simulated hydrothermal degradation induced any phase transformation detectable within the limits of the qualitative XRD analysis used in this study.

## 4. Discussion

The present study evaluated the effects of restorative surface adjustment and simulated hydrothermal aging on the microstructure and biaxial flexural strength of zirconia ceramics with different yttria contents. The results demonstrate that the zirconia composition plays a critical role in determining mechanical performance, microstructural stability, and the response of the material to surface modification procedures. Among the evaluated materials, conventional 3Y‐TZP exhibited the highest biaxial flexural strength, whereas 6Y‐PSZ showed intermediate values and 5Y‐PSZ demonstrated comparatively lower strength.

Surface adjustment performed by grinding with a 100 µm diamond bur, followed by sequential polishing using a zirconia polishing system, significantly increased the flexural strength of both 3Y‐TZP and 5Y‐PSZ. In contrast, the mechanical performance of 6Y‐PSZ remained largely unchanged after the same surface adjustment procedure. XRD analysis of all zirconia groups following the recommended surface adjustment protocol revealed no detectable monoclinic phase formation, indicating that the procedure did not induce any substantial tetragonal‐to‐monoclinic phase transformation within the limits of the qualitative analysis. Furthermore, accelerated hydrothermal aging produced only minor and statistically nonsignificant changes in surface roughness, surface hardness, and biaxial flexural strength across all zirconia groups. Consistent with these mechanical findings, XRD patterns obtained after aging did not reveal detectable phase transformation under the experimental conditions employed in this study.

The differences in mechanical behavior observed among the zirconia materials evaluated in this study can be explained primarily by variations in the phase composition and grain characteristics associated with the amount of yttria used for stabilization. As the yttria concentration increases, the proportion of the cubic zirconia phase progressively rises. It is well established that conventional 3Y‐TZP possesses a microstructure predominantly composed of tetragonal grains, whereas zirconia with a higher yttria content, such as 5Y‐PSZ and 6Y‐PSZ, contains a substantially greater fraction of the cubic phase [23].

These compositional differences are also reflected in the tetragonality of the tetragonal zirconia phase. Materials with lower yttria content typically present a higher tetragonality ratio, indicating that the tetragonal phase is less thermodynamically stable. Although reduced phase stability might appear disadvantageous, it actually facilitates the stress‐induced tetragonal‐to‐monoclinic transformation that underlies the transformation‐toughening mechanism. When a crack develops, this phase transformation produces a localized volumetric expansion that generates compressive stresses around the crack tip, thereby resisting crack propagation and improving fracture resistance [23–25].

In contrast, increasing the yttria content stabilizes the zirconia structure and promotes the formation of the cubic phase, which does not undergo stress‐induced transformation. Consequently, the contribution of transformation toughening is significantly reduced. As a result, zirconia materials containing higher yttria concentrations generally exhibit lower flexural strength, fracture toughness, and fatigue resistance compared with conventional 3Y‐TZP [21, 26–28].

In addition to phase composition, the mechanical performance of dental zirconia is also influenced by microstructural characteristics of the grains, including grain size, grain distribution, and crystallographic structure. These factors affect the crack propagation behavior and stress distribution within the ceramic matrix [29]. Therefore, the combined effects of phase composition and microstructural architecture ultimately determine the overall mechanical properties of zirconia materials with different yttria contents.

A major finding of the present study was the substantial increase in biaxial flexural strength observed in 3Y‐TZP after FP. The polished specimens demonstrated strength values approximately 40% higher than those of the untreated controls. This strengthening effect can be explained by several microstructural mechanisms associated with the surface modification of brittle ceramics.

**Figure 4:**
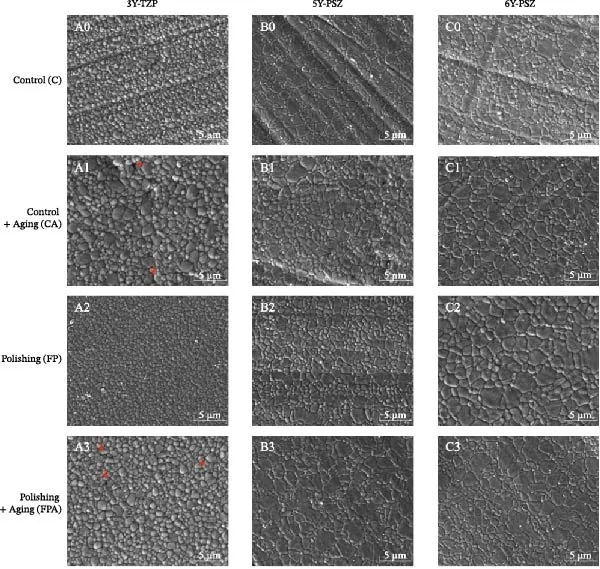
SEM micrographs showing the surface morphology of control group of 3Y‐TZP (A0), 5Y‐PSZ (B0), and 6Y‐PSZ (C0). The simulation of hydrothermal degradation induced the obvious formation of microcrack at grain boundary in 3Y‐TZP (A1 at pointed arrows) control group after aging of 3Y‐TZP (A1), finishing and polishing of 3Y‐TZP (A2), finishing and polishing after aging of 3Y‐TZP (A3), control group of 5Y‐PSZ (B0), control group after aging of 5Y‐PSZ (B1), finishing and polishing of 5Y‐PSZ (B2), finishing and polishing after aging of 5Y‐PSZ (B3), control group of 6Y‐PSZ (C0), control group after aging of 6Y‐PSZ (C1), finishing and polishing of 6Y‐PSZ (C2), and finishing and polishing after aging of 6Y‐PSZ (C3). Arrows point to the surface voids and microcracks.

One important factor contributing to the improvement in strength is the reduction of surface defects. Zirconia restorations fabricated by CAD/CAM milling commonly present machining‐related irregularities such as grooves, scratches, and microcracks on the restoration surface, as illustrated in Figure 4: A0, B0, and C0. These defects act as stress concentrators that facilitate crack initiation when the material is subjected to tensile loading [30]. In brittle ceramics, fracture behavior is strongly governed by the size and distribution of such surface flaws, as described by Griffith’s fracture theory, which states that fracture strength is largely determined by the critical flaw population present at the material surface [15, 31, 32].

FP procedures can effectively remove superficial imperfections and smooth the zirconia surface, thereby reducing surface roughness and decreasing the size of critical surface defects. As a consequence, a greater external load is required to initiate and propagate cracks within the material [15, 33]. This reduction in flaw severity therefore contributes to the observed increase in the flexural strength of 3Y‐TZP after surface finishing procedures.

Another mechanism that may contribute to the strengthening effect involves the generation of compressive residual stresses at the zirconia surface during grinding and polishing. Mechanical surface treatments using diamond burs and polishing abrasives apply localized stresses to the ceramic surface. These stresses may produce limited plastic deformation and microfracture within the superficial grain layer, resulting in distortion of the crystal lattice within the outermost micrometers of the material [34]. In tetragonal zirconia, such deformation may induce micro‐strain within the crystal lattice or stress‐assisted rearrangement of tetragonal grains. The presence of compressive residual stresses is beneficial because it counteracts tensile stresses generated during loading and retards crack initiation and propagation, thereby increasing fracture resistance [32].

XRD observations in the present study support this interpretation (Figure 3: A3, B3, and C3). Following grinding and polishing, the principal diffraction peaks remained located at the same angular positions across all zirconia compositions. Within the limitations of qualitative analysis, this suggests that the primary crystallographic phase structure of the bulk material remained stable [35]. However, slight peak broadening was observed in the magnified region between 28° and 36° (2θ). While quantification of the full width at half‐maximum (FWHM) was not performed, such peak broadening in XRD patterns is commonly associated with lattice microstrain or localized crystallographic distortion rather than phase transformation [36]. This finding suggests the possibility that the finishing procedures introduced residual lattice strain within the superficial layer of the zirconia surface, which could potentially contribute to the altered mechanical behavior without inducing substantial alterations to the bulk crystal structure. Similar observations have been reported in previous studies, suggesting that mechanical surface treatments primarily introduce surface strain and residual stresses while maintaining the stability of the primary crystallographic framework of zirconia [31, 32].

A comparable, although less pronounced, strengthening effect was also observed for 5Y‐PSZ after FP. Unlike 3Y‐TZP, high‐translucency zirconia containing 5 mol% yttria possesses a lower fraction of transformable tetragonal grains and a higher proportion of the cubic phase. Because the cubic phase is crystallographically stable and does not undergo stress‐induced transformation, the transformation‐toughening mechanism that enhances crack resistance in conventional 3Y‐TZP is substantially reduced in 5Y‐PSZ. Consequently, the increase in strength after polishing is likely attributable primarily to surface smoothing and reduction of machining‐induced defects rather than to transformation‐related toughening [37, 38].

In contrast to 3Y‐TZP and 5Y‐PSZ, the biaxial flexural strength of 6Y‐PSZ remained relatively unchanged after FP. This behavior can be explained by the microstructural characteristics of very high‐yttria zirconia. Materials containing approximately 6 mol% yttria exhibit a predominantly cubic microstructure with only a limited fraction of tetragonal grains [39]. In addition, the larger grain sizes typically observed in 6Y‐PSZ may influence fracture behavior by reducing grain boundary density and altering crack propagation pathways [40]. Consequently, although polishing improved the surface smoothness in 6Y‐PSZ, it did not significantly influence the overall fracture strength of the material.

Furthermore, the findings of the present study indicated that the restorative adjustment protocol, consisting of grinding with a 100 µm diamond bur, followed by polishing with a three‐step zirconia polishing system, resulted in a smooth zirconia surface. The measured surface roughness values remained below the clinically accepted threshold of 0.20 μm, which is widely regarded as a critical limit for limiting bacterial plaque adhesion and biofilm formation on restorative materials [41]. Importantly, this favorable surface condition was maintained following simulated hydrothermal aging across all zirconia groups.

Accelerated hydrothermal aging was employed to simulate LTD of zirconia in a humid environment. Specimens were subjected to autoclave aging at 134°C under 0.2 MPa for 5 h, a protocol widely used to reproduce long‐term hydrothermal exposure. Previous studies have suggested that approximately 1 h of autoclave aging under these conditions corresponds to about 3–4 years of clinical service, allowing in vitro simulation of long‐term degradation within a practical experimental timeframe [42].

Despite this accelerated aging protocol, the present results demonstrated that the biaxial flexural strength did not significantly change in any zirconia group after hydrothermal aging. Likewise, XRD analysis did not reveal detectable tetragonal‐to‐monoclinic phase transformation following either FP or the combined effect of surface adjustment and hydrothermal aging. One possible explanation relates to the surface‐limited nature of the hydrothermal degradation in zirconia. LTD begins with the penetration of water molecules into the zirconia lattice, which promotes the transformation of metastable tetragonal grains into the monoclinic phase. This transformation typically initiates at the material surface and gradually progresses toward the interior [43].

Notably, while qualitative XRD analysis showed no detectable phase transformation, SEM observations of the aged 3Y‐TZP revealed minor microstructural alterations, including grain‐boundary microcracking and localized grain pull‐out. This discrepancy suggests that although the volume fraction of the transformed monoclinic phase remained below the detection limit of qualitative XRD, localized hydrothermal degradation had nonetheless been initiated at an early, microstructural level. Consequently, while these early microstructural changes did not significantly compromise the biaxial flexural strength, they reveal the early onset of aging effects that could potentially influence the long‐term clinical performance.

Because biaxial flexural strength is primarily governed by the overall structural integrity of the bulk material, localized surface alterations may not substantially affect the measured strength unless degradation penetrates deeper into the ceramic structure. Previous studies have reported similar findings, showing that hydrothermal aging may induce monoclinic phase formation at the surface without significantly altering the bulk mechanical properties [44]. Consequently, although LTD may modify certain surface characteristics such as roughness, grain pull‐out, or susceptibility to microcracking, the overall flexural strength of zirconia restorations may remain largely unaffected when transformation is restricted to a superficial region [45, 46].

These interpretations are consistent with the fractographic observations obtained in the present study. All zirconia materials exhibited typical brittle fracture characteristics regardless of the surface treatment or aging condition. Although minor variations in surface roughness and fracture morphology were observed, particularly in 3Y‐TZP, no distinct morphological features indicative of severe surface degradation or microstructural damage were detected after finishing, polishing, or hydrothermal aging. This observation further supports the conclusion that the applied treatments primarily influenced the surface condition of the specimens without compromising the bulk structural integrity of the zirconia materials.

Nevertheless, some limitations of the present study should be considered. The experimental design was conducted under controlled laboratory conditions, which cannot fully replicate the complex intraoral environment where restorations are subjected to cyclic occlusal loading, temperature fluctuations, pH changes, and biological interactions. In addition, the mechanical evaluation was limited to biaxial flexural strength, and other important properties such as fatigue resistance and slow crack growth behavior were not investigated. Future research should further investigate the long‐term durability of zirconia with different yttria contents under thermomechanical fatigue conditions that more closely simulate the oral environment.

## 5. Conclusion

Within the limitations of this in vitro study, the zirconia composition and yttria content significantly influenced the microstructure and biaxial flexural strength of the investigated materials. Conventional 3Y‐TZP exhibited the highest strength, whereas 5Y‐PSZ showed the lowest values, and 6Y‐PSZ demonstrated an intermediate mechanical performance. These differences are consistent with variations in phase composition and grain characteristics associated with the increasing yttria content.

Restorative surface adjustment using a 100 µm diamond bur followed by a three‐step zirconia polishing system significantly increased the biaxial flexural strength of 3Y‐TZP and, to a lesser extent, 5Y‐PSZ, while 6Y‐PSZ remained unaffected. The strengthening effect observed in 3Y‐TZP and 5Y‐PSZ is likely associated with the removal of surface defects and reduction of critical flaw size, together with the possible development of beneficial compressive residual stresses at the surface. XRD analysis confirmed that the adjustment and polishing procedures did not induce detectable tetragonal‐to‐monoclinic phase transformation.

Under the applied accelerated hydrothermal aging conditions, no significant changes in bulk flexural strength, surface roughness, surface hardness, or crystallographic phase composition were detected in any zirconia group. However, SEM observations revealed early surface microstructural changes in 3Y‐TZP consistent with the initial LTD, suggesting that longer‐term aging or fatigue studies may be warranted before broad conclusions regarding clinical durability can be drawn.

Overall, the findings indicate that clinically relevant occlusal adjustment followed by appropriate zirconia polishing does not adversely affect the mechanical performance of contemporary zirconia ceramics and may even enhance the strength of conventional 3Y‐TZP. These results support the clinical recommendation that zirconia restorations undergoing intraoral adjustment should be carefully polished to restore a smooth surface, thereby maintaining immediate mechanical reliability while minimizing the potential for long‐term microstructural degradation.

## Author Contributions

Conceptualization, writing – review and editing: Phawinee Phuntusuntorn, Kullapop Suttiat, and Natthawat Pleumsamran. Methodology, validation, formal analysis: Phawinee Phuntusuntorn and Kullapop Suttiat. Investigation, resources, data curation, writing – original draft, visualization, project administration, funding acquisition: Phawinee Phuntusuntorn. Supervision: Kullapop Suttiat and Natthawat Pleumsamran.

## Funding

This work was supported by the Faculty of Dentistry, Chiang Mai University.

## Disclosure

All authors have read and approved the final version of the manuscript. The corresponding author had full access to all of the data in this study and takes complete responsibility for the integrity of the data and the accuracy of the data analysis. All authors contributed significantly to the reported work, participated in drafting or revising the manuscript, and agree to be accountable for all aspects of the study. The funding source had no role in the study design; in the collection, analysis, or interpretation of data; in the writing of the manuscript; or in the decision to publish the results.

## Ethics Statement

This study is an in vitro laboratory investigation using dental zirconia materials and did not involve any human participants, biological tissues, or animal subjects. Consequently, institutional ethical approval was not required.

## Conflicts of Interest

The authors declare no conflicts of interest.

## Data Availability

The data that support the findings of this study are available from the corresponding author upon reasonable request.
